# Transpedicular Transdural Approach for Calcified Thoracic Disc Herniations: Technical Commentary, Case Series, and Review of the Literature

**DOI:** 10.3390/jcm14217741

**Published:** 2025-10-31

**Authors:** Spyridon Komaitis, Elie Najjar, Dritan Pasku, Konstantinos Zygogiannis, Daniel D’Aquino, Khalid M Salem

**Affiliations:** The Centre for Spinal Studies and Surgery, Queen’s Medical Centre, Nottingham NG7 2UH, UK; elie.najjar@nhs.net (E.N.); n.paskou@nhs.net (D.P.); konstantinos.zygogiannis@nhs.net (K.Z.); daniel.daquino@nhs.net (D.D.); khalid.salem2@nhs.net (K.M.S.)

**Keywords:** calcified thoracic disc, transdural approach, thoracic discectomy, spinal cord decompression, microsurgical technique

## Abstract

**Background/Objectives**: Calcified thoracic disc herniations are a formidable surgical challenge due to their proximity to the spinal cord and the heightened risk of iatrogenic neurological injury. Traditional anterior and posterolateral approaches, while effective in select scenarios, may not provide adequate exposure for large, centrally located calcified discs. **Methods**: We performed a narrative review of the literature and retrospective case series of seven patients who underwent transpedicular–transdural thoracic discectomy for central or centrolateral calcified disc herniations at our institution in recent years. All patients were followed clinically for a minimum of three months postoperatively. Surgical technique and intraoperative nuances were also documented. **Results**: The transdural approach enabled direct access to the ventral thoracic spine, allowing for the effective decompression of calcified herniations in all cases. Six out of seven patients (86%) demonstrated clinical improvement or neurological stability at three-month follow-up, while one out of seven patients (14%) who presented with severe preoperative neurological deficits had persisting neurological deterioration postoperatively. The technical aspects of the microsurgical approach were critical to minimizing risk. **Conclusions**: The transpedicular–transdural approach is a viable and effective surgical option for select cases of central or centrolateral calcified thoracic disc herniation. When executed with a precise microsurgical technique, it offers safe decompression with favourable short-term outcomes.

## 1. Introduction

Thoracic disc herniations (TDHs) are rare entities, accounting for fewer than 1% of all symptomatic disc herniations, and they often occur in the thoracolumbar junction [1,2,3]. Surgical management becomes necessary mainly in cases of progressive myelopathy, but it can also be necessary in the presence of disabling radiculopathy or severe axial pain with radiological evidence of cord compression [4]. Calcified TDHs, particularly those located centrally or centrolaterally, are technically demanding to remove due to their adherence to the dura and the close proximity of the thoracic spinal cord, which is less tolerant to manipulation compared with the cervical and lumbar regions.

A variety of surgical approaches have been described, each with distinct benefits and drawbacks [5,6]. Anterior approaches, including open transthoracic and minimally invasive thoracoscopic discectomy, allow for the direct visualization of the ventral disc space and facilitate safe decompression, especially in cases with extensive calcification. However, these techniques are associated with increased perioperative morbidity, longer operative time, and the need for access to surgeons or specialized equipment, particularly in cases of high thoracic levels [7,8]. Posterolateral approaches, such as costotransversectomy or lateral extracavitary techniques, aim to balance exposure with a less invasive corridor. These approaches provide lateral or oblique access to the disc space while avoiding direct manipulation of the spinal cord, but they also often offer suboptimal visualization of centrally located herniations, particularly when calcified [9,10].

The posterior transdural approach, though less frequently utilized, provides a direct intradural corridor to centrally or centrolaterally located disc fragments. This technique allows for controlled retraction of the spinal cord within the dural sac and direct microsurgical removal of the disc under high magnification [11,12]. Despite its theoretical advantages, transdural discectomy is technically demanding and associated with specific risks, including cerebrospinal fluid (CSF) leak, arachnoid scarring, and injury to the spinal cord or vascular structures. As a result, its use has remained limited; it is typically reserved for select cases where it can be performed by experienced hands [13,14].

At our institution, transdural thoracic discectomy is selectively performed for centrally or centrolaterally located large calcified TDHs in cases where other approaches are unlikely to provide safe and complete decompression. In this report, we present a series of seven patients treated over the past year with a transdural approach, and we provide a detailed technical commentary of the surgical recommendations and considerations that guide our operative strategy.

## 2. Materials and Methods

Between May 2024 and May 2025, seven patients with large, symptomatic, centrally or centrolaterally located calcified thoracic disc herniations underwent transdural thoracic discectomy at our institution. The indication for following a transdural route was the presence of a large or giant central or centrolateral calcified thoracic disc (occupying >40–50% of the canal cross-section). In such cases, using a posterolateral extradural approach might be limited in terms of poor visualization of the pathology, and using a transthoracic approach might result in the presentation of obvious risks due to the presence of severe adherence to the dura and a very high risk of intrathoracic CSF leak. All procedures were performed by a group of two of the authors (K.S. and S.K or K.S and D.D.) using a standardized microsurgical technique. All patients underwent a comprehensive clinical and radiological workup, including thoracic spine magnetic resonance imaging and high-resolution thoracolumbar computed tomography. MRI was primarily used to assess spinal cord compression, myelomalacia, and the degree of canal compromise, while CT imaging was essential to characterize the extent, density, and distribution of disc calcification. CT was also utilized to delineate bony anatomy and plan the optimal surgical trajectory. Rib counting on preoperative CT with anteroposterior reconstructions was routinely employed to ensure accurate vertebral level localization and avoid wrong-level surgery, which is a known risk in thoracic spine procedures due to the potentially variable rib anatomy and difficulties with intraoperative fluoroscopy. Postoperatively, patients were monitored in a high-dependency unit and mobilized as tolerated by each patient. Neurological examinations were performed immediately after surgery and during routine clinical follow-up at 1 week, 1 month, and 3 months. Functional outcome measures (Nurick and Frankel scores) were recorded systematically by two of the authors (K.S. and S.K.) to ensure inter-observer reliability. Follow-up imaging was performed selectively based on clinical indication. Anonymization of all photographic material was meticulously performed, and relevant consent was obtained from all patients for their images to be published. Following the presentation of our case series, we proceed with a narrative review of the pertinent literature. PubMed studies focusing on the treatment of patients with thoracic disc herniations by means of transdural discectomy have been included. Main emphasis has been placed on the surgical technique utilized and the neurological outcomes.

## 3. Results and Surgical Technique

Seven patients (four males and three females; mean age 58.3 years, range 47–69) underwent transdural thoracic discectomy for centrally or centrolaterally located calcified thoracic disc herniations. All procedures were performed with the aid of intraoperative neuromonitoring and image-guided navigation. The mean operative time was 241 min (range: 217–263 min), and the mean estimated blood loss was 385 mL (range: 280–520 mL). Preoperatively, the mean Nurick grade was 3.9 (range: 3–5), reflecting moderate-to-severe thoracic myelopathy. At 3-month follow-up, six of the seven patients improved neurologically, with a reduced mean Nurick grade of 2.4. Patient 4 initially presented with severe myelopathy and paraparesis, power of 2/5, in both lower limbs and has been bedbound for 3 months prior to admission. Intraoperatively and during laminectomy, a loss of motor evoked potentials of more than 80% was observed. Postoperatively, the patient presented with severe paraparesis (motor score of 1/5 in most muscle groups of the lower limbs) but preserved a light touch sensation. No motor recovery was observed at the 3-month follow-up. Patient 5 remained neurologically stable immediately postoperatively but developed transient paraparesis on postoperative day two after a hypotensive episode on the ward. An emergency MRI was performed, but this was limited by a metal artefact and was inconclusive for compressive pathology. Given their clinical deterioration, the patient underwent re-exploration within three hours, which revealed that there was no compressive collection. Postoperatively, the patient was managed in the high-dependency unit with blood pressure augmentation, leading to full neurological recovery within 24 h. There were no CSF leaks through the wound, and no wound infections were recorded. No hardware-related complications were observed. No cases of early-onset adhesive arachnoiditis were observed, although delayed arachnoiditis needs to be investigated with longer follow-up.

**Figure 1:**
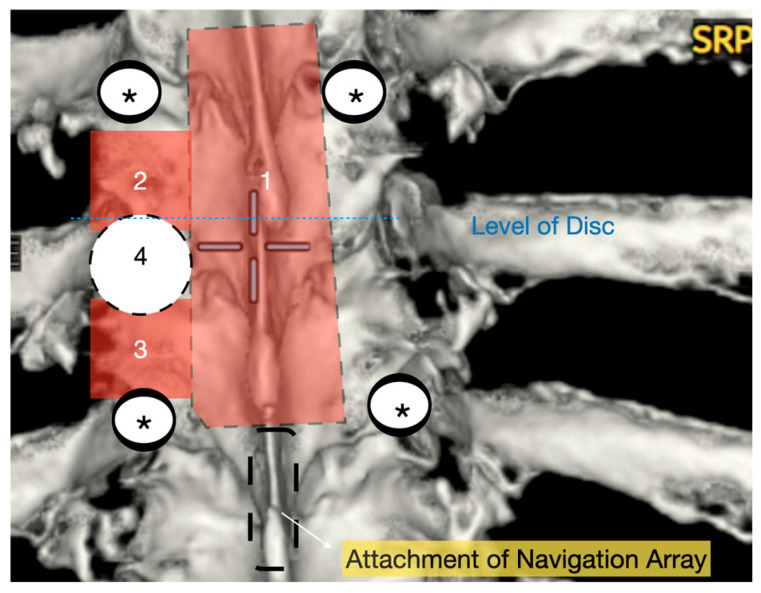
Three-dimensional reconstruction of area of interest (T9–11) with representation of osteotomies carried out with bone scalpel. 1: En block laminectomy; 2: ipsilateral upper facetectomy; 3: ipsilateral lower facetectomy; 4: ipsilateral pediculectomy. *: screws.

Patients were positioned prone on an Allen Table with chest bolsters and head stabilization using a Mayfield clamp. Intraoperative neuromonitoring (MEPs and SSEPs) and neuronavigation (O-Arm with StealthStation) were employed in all cases. A midline incision was made according to the preoperative rib counting and extended caudally by one level below the lowest planned instrumentation to allow for secure placement of the navigation reference array (Figure 1). An initial O-Arm spin was acquired, and pedicle screws were placed one level above and two levels below the herniated disc. The pedicles immediately inferior to the disc space were deliberately left uninstrumented to facilitate ipsilateral pediculectomy and optimize access to the ventral spinal canal.

**Figure 2:**
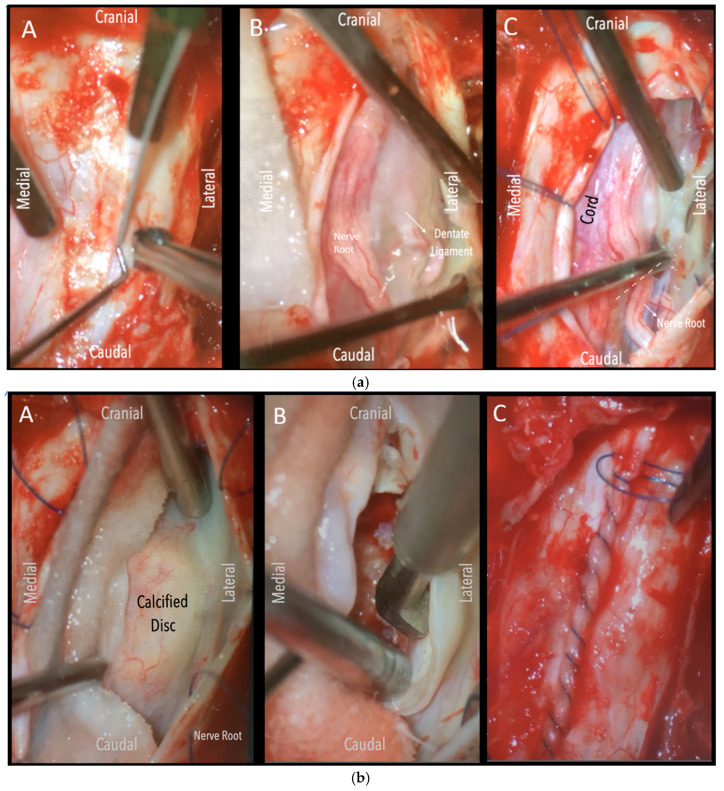
(**a**)**.** Surgical field under microscope magnification. A: Opening of dura with no. 15 blade. B: Release of CSF. Following this, the table was tilted contralateral to the disc side to allow the cord (see annotation in image on the left) to slowly drift away. The dentate ligament, appearing as a thickening of the arachnoid, can be identified and dissected with microscissors. C: The nerve root is evident. If required, it could be sacrificed intramurally (see dashed line and annotation in image on the left side) with microscissors after being coagulated with bipolar diathermy. (**b**)**.** Intraoperative microscope view of the same case. Right-sided disc. A: The calcified disc is evident after gentle gravity-retraction of the cord. B: A hockey stick bone scalpel (MISONIX). C: Watertight closure of dorsal dura.

**Figure 3:**
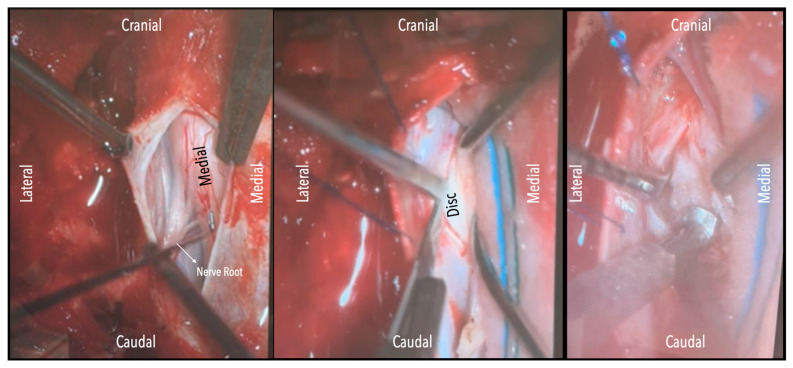
Intraoperative microscope view. Left-sided disc. Left: After opening the dura mater, the nerve root and cord are evident. Middle: Following drainage of CSF, dissection of the dentate ligament and gravity retraction, a micro-pattie is applied on the lateral aspect of the cord and a penfield microdissector is used to gently retract the cord, and the disc becomes evident. Right: Discectomy is carried out with a hockey stick bone scalpel.

A wide en bloc laminectomy was performed at the disc level and one level above and below using a Misonix bone scalpel. This was followed by ipsilateral superior and inferior facetectomies and complete pediculectomy to establish an oblique and spacious working corridor toward the ventral spinal canal (Figure 1). Under the operating microscope, the dura was opened in the midline with a no. 15 blade, and the arachnoid was incised sharply. CSF was allowed to drain passively for 10–15 min to reduce intradural pressure and promote relaxation of the spinal cord. The table was then tilted approximately 6–10 degrees to the contralateral side to allow for gravity-assisted displacement of the cord away from the operative field (Figure 2a,b and Figure 3).

**Figure 4:**
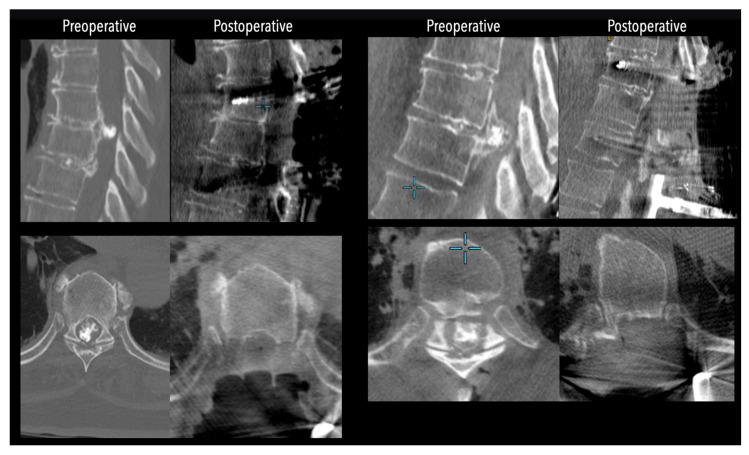
Comparative pre- and postoperative CT scans for two different patients (left and right), illustrating complete discectomy.

**Figure 5:**
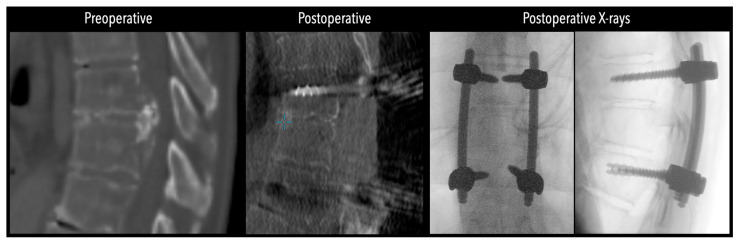
Left side: Comparative pre- and postoperative CT scan of a patient, illustrating complete discectomy. Right side: Postoperative X-rays illustrating fixation.

**Figure 6:**
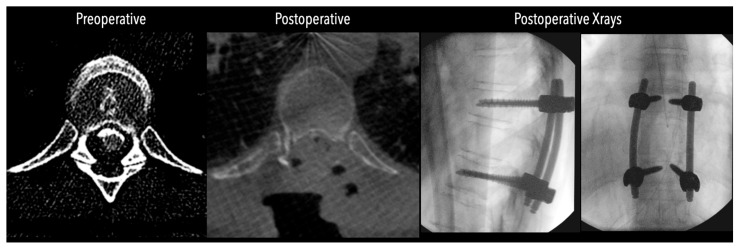
Left side: Comparative pre- and postoperative CT scan of a patient (axial views), illustrating complete discectomy.

In three of the seven cases, the exiting nerve root at the level of the herniation was ligated due to poor visualization and transected extradurally to improve access. In the remaining four patients, the nerve root was either preserved or dissected intradurally using isocool bipolar diathermy and microsurgical scissors. With gentle medial retraction of the cord under gravity and protection using a micro-patty, the calcified disc was exposed. The ventral dura, when intact, was incised sharply and dissected from the disc surface where possible (Figure 2a,b and Figure 3). Disc removal was performed using a hockey-stick-shaped Misonix bone scalpel with constant irrigation, guided by intraoperative neuronavigation. The disc material was resected to a depth of 1–2 mm beyond the posterior vertebral wall to ensure adequate decompression. A second O-Arm spin was routinely performed following discectomy to confirm satisfactory decompression, especially given the limitations of the postoperative MRI due to the metal artefact (Figure 4, Figure 5 and Figure 6).

Extensive irrigation of the intradural and extradural spaces was performed to minimize the risk of postoperative adhesive arachnoiditis. In all cases, the ventral dural defect was patched with TachoSil. The dorsal dura was closed primarily with a watertight suture using interrupted 5-0 Prolene and additionally patched with TachoSil. A gravity-dependent deep wound drain was routinely left in place for four days postoperatively. After this period, the drain was clamped, and the wound was monitored for 12 h. Once we ensured that there was no CSF leakage, the drain was removed, and the exit site was closed. Patients were monitored in the high-dependency unit (HDU) for 24–48 h postoperatively. Mean arterial pressure (MAP) was maintained at ≥85 mmHg for 48 h.

In cases of intraoperative loss of monitoring with a drop in postoperative neurology, we routinely proceed with mean arterial pressure augmentation (>85 mmHg) for 5 days and flat bed rest for 48 h to facilitate cord perfusion. Mobilization was initiated on the second postoperative day. Once we ensured that there was no neurological deterioration, no further imaging was performed during the hospital stay. All patients were seen three months postoperatively. Clinical assessment included evaluation of neurological function and mobility. Preoperative and postoperative Nurick and modified Frankel scores were recorded to objectively assess functional trajectory. Patient demographics, pathology characteristics, and outcomes are summarized in Table 1. 

## 4. Discussion

Transdural thoracic discectomy remains an underutilized approach, often reserved for select cases due to its technical demands and the perceived risk of intradural manipulation. However, in appropriately chosen patients and when performed by experienced surgeons, it can provide safe and effective decompression for centrally located, calcified thoracic disc herniations. Our experience demonstrates that the transdural approach can be performed with a high safety profile, provided that a meticulous microsurgical technique is employed. The approach is particularly advantageous in cases involving large, centrally positioned calcified discs where posterolateral extradural techniques offer poor visualization and transthoracic approaches carry significant morbidity. The transdural approach combined with bilateral pediculectomies and facetectomies offers the advantage of direct visualization of the calcified disc, drainage of CSF, and transection of the dentate ligament, which allow for a degree of cord manipulation with wide exposure and decompression as opposed to the traditional extradural transfacet or costotransversectomy approaches, where retraction of the dura might be required with frequently very poor visualization of the ventral pathology. On the other hand, transthoracic approaches offer an excellent visualization of the disc area; however, they might present with significant challenges in cases of calcified discs that are severely adherent to the ventral dura. In such cases, an intrathoracic CSF fistula frequently occurs, leading to high morbidity, the need for long ICU admission, or prolonged chest drain treatment.

In our series, one out of seven patients experienced lasting neurological deterioration (14.3%). Although the risk of neurological deterioration following thoracic discectomy remains vague in the literature, this number is comparable to the rates of neurological complications reported in other series, where deficits have been reported in 5–23% of cases, particularly in calcified or centrally located disc herniations [7,15].

We found that the exposure afforded by this approach is excellent. Following the release of the dentate ligament and tilting the table, which allows for gravity-assisted cord retraction, a generous and safe working corridor was created. In cases where access was limited, sacrificing the exiting nerve root was necessary in a minority of patients; however, we advocate for intradural dissection of the nerve root when possible, as extradural ligation may compromise radiculomedullary arterial supply, potentially precipitating spinal cord ischemia. Additionally, performing en bloc laminectomy and facetectomies using ultrasonic tools such as the Misonix bone scalpel was not only safe in our hands but also helpful to reduce operative time and minimize the manipulation of surrounding structures. One of the most technically challenging aspects of these cases is the ventral dura, which is often densely adherent to the calcified disc and fibrotic. In such settings, complete and safe discectomy via posterior–lateral or anterior approaches may be difficult, if not impossible. The transdural route offers direct midline access that facilitates more thorough decompression without more controlled spinal cord passive retraction in the presence of reduced intradural pressure.

Importantly, no patient in our series experienced CSF-related complications such as persistent leaks or large pseudomeningocele. We attribute this to meticulous dorsal dural closure, ventral dural patching, and careful postoperative wound and drain management. Nonetheless, longer-term follow-up is essential, as late-onset adhesive arachnoiditis remains a potentially devastating complication of intradural spinal surgery and may not be evident during the early postoperative period.

The available literature provides evidence that, through implementing a posterior approach, adequate decompression concerning thoracic calcified discs can be achieved. The results of a total of 328 patients were described in 11 articles examining their clinical efficacy, safety profile, and surgical outcomes in comparison with other established techniques [16]. The demographic profiles of the patients across the pooled studies aligns closely with those reported in the literature involving alternative surgical methods for TDH. The mean age of the patients was 50.19 ± 5.54 years, with a nearly equal gender distribution, with 51.5% male and 48.5% female. The most frequent clinical presentations were myelopathy and radiculopathy, and the majority of disc herniations occurred below the T6–7 level [17,18]. Although multilevel herniation was historically considered rare, improvements in imaging technology have led to increased detection rates. In the analyzed studies, multilevel disc herniation was reported in 38% of patients (36 out of 95), reflecting a shift in the understanding of TDH presentation patterns [19,20].

While the anterior transthoracic approach is typically favoured for addressing central and calcified thoracic discs, its invasiveness presents notable drawbacks, including increased cardiopulmonary risks due to rib resection and pleural disruption. The transfacet pedicle-sparing approach, as originally proposed by Stillerman et al., involves preserving the lateral facet to maintain spinal stability and minimize postoperative axial back pain. However, several studies in this analysis employed a modified version of the technique that involves complete facetectomy. This modification was implemented to improve access to the disc space by creating a wider diagonal surgical corridor, thereby reducing the need for excessive dural retraction. To address the resulting spinal instability from facetectomy, instrumentation and segmental fusion were performed in these cases. Among the 176 herniated discs analyzed, 30% were central and 18% were calcified, suggesting the adaptability of this modified posterior approach to a wide range of disc morphologies [21].

Clinical outcomes from the included studies demonstrated significant improvements in terms of both pain and neurological function following surgery. The visual analogue scale (VAS) scores, reported across four studies with low heterogeneity (I^2^ = 10%), showed a standardized mean difference (SMD) of 0.749 (95% CI: 0.555–0.943) [19,20], indicating meaningful pain reduction. Neurological improvement was also observed, as evidenced by Nurick scores from five studies, yielding an SMD of 0.775 (95% CI: 0.479–1.071) despite moderate heterogeneity (I^2^ = 64.4%) [19,20,22]. These findings underscore the potential benefits of the transfacet pedicle-sparing approach in achieving adequate spinal cord decompression while minimizing the risks of aggressive cord manipulation and extensive soft tissue dissection.

The postoperative complication rates associated with the posterior approach were notably lower than those reported for anterior approaches. In this analysis, eight studies documented a combined complication rate of 12.4% (32 out of 258 patients), with only 3.5% (9 patients) experiencing neurological deterioration. These figures compare favourably with previous reviews, such as that by Hurley et al., which reported a 23% complication rate for anterior procedures versus 14% for posterior approaches. Similarly, Yoshihara et al. identified a 26.8% complication rate for anterior surgeries using national registry data [23]. In our study, 1/7 (14%) patients developed persistent neurological deterioration. This was identified as a sudden loss of MEPs (SSEPs have been absent throughout the procedure) intraoperatively that remained throughout the follow-up period. The above loss of MEPs occurred during posterior decompression, before the transdural stage, and might be related to the use of the bone scalpel in a very narrow canal. In such cases, in the presence of severe canal stenosis, it would be advisable to perform the decompression with extreme caution using a burr or a piece-meal technique if deemed necessary. It is important to stress that the above patient had a very poor baseline with significantly low motor scores and had been bedbound for at least 3 months preoperatively.

The relatively lower morbidity associated with the transfacet pedicle-sparing approach may contribute to shorter hospitalizations; in this study, four studies reported an average hospital stay of 4.6 days across 166 patients. Additionally, five studies reported an average intraoperative blood loss of 580 cc and a mean follow-up duration of 13.8 months, with five patients lost to follow-up [23,24]. The decision to perform spinal fusion following thoracic discectomy remains a topic of debate. While fusion is generally reserved for cases involving giant calcified discs, spinal deformity, or multilevel procedures, some studies advocate for its routine use to prevent postoperative instability, particularly in the lower thoracic spine, where mechanical flexibility is greater. In this analysis, fusion practices varied. Four studies reported fusion even for single-level herniations, one study reserved fusion for cases involving three or more consecutive levels, and three studies performed no fusion despite complete facetectomies. These inconsistencies highlight the need for long-term investigations to clarify whether total facetectomy without fusion predisposes patients to spinal instability or other complications. The interplay between thoracic vertebrae, discs, the rib cage, and sternum contributes to the inherent stability of the thoracic spine, making the performance of fusion a context-dependent decision.

In their study, Coppes et al. reported that between September 2004 and October 2010, 13 patients with symptomatic central thoracic disc herniation underwent surgery using a posterior transdural approach. Preoperative and postoperative assessments included MRI, Frankel scores, and patient interviews using a seven-point Likert scale. The most commonly affected levels were T10–11 and T12–L1. The median operative time was 210 min, and the median hospital stay was 6 days. In our study, the median operative time was 245; however, in our cohort, fixation was performed in all cases, as opposed to Coppes et al.’s study. Three patients experienced reversible complications, and no spinal fixation was required. With a median follow-up of 18 months, 92% of patients showed symptom improvement (71% in our study), while one patient remained unchanged, and none worsened (0% vs. 14% in our study). The difference in the number of patients that showed long-term improvement might be attributed to the short follow-up period of our study [11].

In their study in 2023, Doğan et al. reported a case series of seven patients who underwent transdural, facet, and nerve-root-sparing discectomy for symptomatic thoracic disc herniation. Five out of the seven patients presented with calcified discs, whereas two had soft disc herniations. The affected levels ranged from T7–T8 to L1–L2. The authors reported a minimum follow-up period of six months, during which all patients (100%, compared with 86% in our series) demonstrated neurological stability or improvement [25].

In 2020, Negwer et al. presented a case series of 12 patients who underwent posterior transdural resection of calcified thoracic disc herniations between 2012 and 2020. In this cohort, four patients experienced immediate postoperative neurological deterioration, and two (16%, compared with 14% in our series) were discharged with worsened mJOA scores. At long-term follow-up, 11 out of 12 patients (92%, compared with 86% in our study) demonstrated neurological stability or improvement relative to their preoperative baseline. The shorter follow-up period in our study may partly account for this discrepancy [26]. We present a summary of the current available literature and outcomes for patients undergoing transdural thoracic discectomy in Table 2.

Overall, thoracic discectomies are relatively rare procedures in clinical practice, largely due to the unique anatomical and biomechanical characteristics of the thoracic spine. Among the various surgical techniques, the transdural approach represents an uncommon yet valuable corridor in selected cases, particularly when dealing with large, centrally located or calcified disc herniations. However, the literature on this technique remains very limited, with most reports consisting of small case series or technical descriptions. Therefore, further studies are needed to systematically evaluate the safety, efficacy, and long-term outcomes of the transdural approach.

## 5. Limitations

The primary limitation of this study is its small sample size. However, transdural thoracic discectomy remains an exceptionally rare and technically advanced procedure, with only limited case series published in the literature to date. Thus, we believe that our findings contribute meaningful insight into this niche but important area of spinal surgery. The study period of one year limits the generalizability of the conclusions that can be extrapolated, and long-term follow-up is necessary to monitor for delayed complications such as adhesive arachnoiditis or ventral cord herniation, which may not become clinically evident for several months or even years. It must be emphasized that outcomes with this approach are closely tied to the operating surgeon’s experience and familiarity with complex intradural microsurgical techniques, limiting generalizability to centres without such expertise. Finally, the introduction of modern techniques like spinal endoscopy and robotic surgery has offered new insights and might eventually lead to a paradigm shift in the treatment of thoracic disc herniations [27].

## 6. Conclusions

The transdural approach represents a valuable option for the treatment of large, central calcified thoracic disc herniations in selected patients. Our case series offers useful insights into both the technical aspects of the procedure and the short-term clinical outcomes. With appropriate patient selection and a meticulous microsurgical technique, this approach can achieve effective decompression with an acceptable safety profile. Further studies with longer follow-up are needed to better understand the long-term outcomes and risk of delayed complications, such as adhesive arachnoiditis, since the follow-up period of the current study is limited.

## Figures and Tables

**Table 1:** Patient demographics, pathology characteristics, and outcomes.

Patient	G	Age	Pathology	Approach	Ligation of the Nerve	Complete Discectomy Achieved	Preoperative Status	Nurick Grade Preop	Nurick Grade 3 Months Postop	Modified Frankel Score Preop	Modified Frankel Score Postop	IBL	OD	NMA
1	M	55	Calcified Thoracic Disc T9–10	Intradural Discectomy	Yes—Extradural	Yes	Severe myelopathy—mobilizing only with assistance	5	3	3b	4	300	230	No
2	M	37	Calcified Thoracic Disc T9–10	Intradural Discectomy	Yes—Intradural	Yes	Severe myelopathy—mobilizing only with assistance	5	5	3b	3b	250	250	No
3	F	56	Calcified Thoracic Disc T8–9	Intradural Discectomy	Yes—Intradural	Yes	Mild myelopathy—able to mobilize independently	1	1	5	5	400	215	No
4	F	68	Calcified Thoracic Disc T9–10	Intradural Discectomy	Yes—Intradural	Yes	Severe myelopathy with tetraparesis—bedbound for 3 months prior to admission	5	5	3b	2	200	270	Loss of MEPs
5	F	68	Calcified Thoracic Disc T7–8	Intradural Discectomy	No	Yes	Severe myelopathy—mobilizing only with assistance	5	3	3b	5	250	245	No
6	F	63	Calcified Thoracic Disc T7–8	Intradural Discectomy	Yes—Extradural	Yes	Moderate Myelopathy	4	3	4	5	350	250	No
7	M	59	Calcified Thoracic Disc T10–11	Intradural Discectomy	Yes—Extradural	Yes	Moderate Myelopathy	3	3	4	4	150	255	No

G: gender, F: female, IBL: intraoperative blood loss, M: male, NMA: neuromonitoring alert, OD: operative duration.

**Table 2:** Comparison of reported neurological outcomes of transdural thoracic discectomy in the existing literature.

Study	Number of Patients	Duration of Study	Follow-Up	Patients Improving or Remaining Stable in Long Follow-Up	Patients with Neurological Deterioration Immediately Postop.	Patients with Neurological Deterioration in Last Follow-Up
Moon et al. (2010) [13]	3		12 months (minimum)	100% (3 patients)	0% (0 patients)	0% (0 patients)
Coppes et al. (2012) [11]	13	2004–2010	3 months (minimum)	100% (13 patients)	0% (0 patients)	0% (0 patients)
Negwer et al. (2020) [26]	12	2012–2020	303 days (median follow-up)	92% (11 patients)	33% (4 patients)	0 (1 patient did not have follow-up)
Dogan et al. (2023) [25]	7	2012–2020	6 months (minimum)	100% (7 patients)	0% (0 patients)	0% (0 patients)
Current Study	7	2024–2025	3 months	86% (6 patients)	14% (1 patient)	14% (1 patient)

## Data Availability

Data can be provided upon reasonable request via e-mail.

## Abbreviations

TDHs	Thoracic disc herniations
CDF	Cerebrospinal fluid
